# Sampling Schemes in Poliovirus Wastewater Surveillance Studies from European Countries and Their Comparison to Other Studies: A Literature Review

**DOI:** 10.3390/microorganisms14040861

**Published:** 2026-04-11

**Authors:** Jan Rožanec, Veronika Učakar, Andrej Steyer, Rosa M. Pintó Solé, An Galičič

**Affiliations:** 1 National Institute of Public Health, Trubarjeva ulica 2, SI-1000 Ljubljana, Slovenia; 2 National Laboratory for Health, Environment and Food, Prvomajska ulica 1, SI-2000 Maribor, Slovenia; 3 Faculty of Biology, University of Barcelona, Avinguda Diagonal 643, ES-08028 Barcelona, Spain

**Keywords:** poliomyelitis, poliovirus, wastewater-based epidemiology, communicable diseases, sampling schemes

## Abstract

Poliovirus wastewater surveillance (WWS) is an important complementary system to acute flaccid paralysis surveillance and a substitute surveillance for enteroviruses in stool samples of children, within global poliovirus eradication efforts. However, current guidelines provide limited data on sampling schemes for polio-free countries. This study aims to provide a summary of sampling schemes used for poliovirus WWS in studies done in countries of European Union (EU), together with an analysis of their objectives and a comparison with studies done in other countries. The study selection for this literature review was based on three sources: a World Health Organisation literature review, the PubMed database, and a systematic literature review of WWS of communicable disease agents. From 1446 studies, 72 studies published between 1 January 1995 and 5 September 2024 were included in the qualitative analysis. These studies were analysed by country of origin, objective, sampling site, catchment population size, sampling frequency, sampling method and sample volume. The results indicate that most studies from EU countries had conducted poliovirus WWS directly in line with World Health Organisation recommendations, despite these being primarily designed for developing countries. Our review may encourage countries to optimise their poliovirus WWS systems based on their own risks for poliovirus introduction and circulation.

## 1. Introduction

Poliovirus wastewater surveillance (WWS) plays an important role in the efforts of the Global Polio Eradication Initiative (GPEI) [1,2]. In the context of poliovirus eradication, WWS serves as a complementary system to acute flaccid paralysis (AFP) surveillance and a substitute surveillance for enteroviruses in stool samples of children. Epidemiological surveillance of clinical cases enables targeted investigation of poliomyelitis cases and provides the basis for risk assessment of poliovirus transmission at the community level [3,4]. WWS for polioviruses complements this surveillance system by enabling the detection of unidentified infected individuals whose wastewater flows into a common sewer system [5], allowing early detection of “silent” transmission in the population [6,7]. As the global number of poliomyelitis cases continues to decline and most countries are already classified as polio-free, poliovirus WWS is increasingly important [8].

In polio-free countries, poliovirus WWS is important for the early detection of possible wild poliovirus (WPV) reintroduction and emergence of circulating vaccine-derived poliovirus (cVDPV) in the population. In addition, it enables the surveillance of diagnostic laboratories and vaccine production facilities that hold live polioviruses, with monitoring of wastewater from those facilities. In areas with recent poliovirus circulation, the objective of poliovirus WWS is to document the scope of virus transmission to guide immunisation activities and to assess their effectiveness in outbreak response. Additionally, the poliovirus WWS provides relevant information to confirm the end of an outbreak through the absence of WPV or vaccine-derived poliovirus (VDPV) in wastewater samples, together with the negative detection of poliovirus in stools from AFP cases. In endemic countries, the objective of poliovirus WWS is to monitor circulating polioviruses and their genetic variants, while complementing AFP surveillance by identifying epidemiological links between geographical areas, potential virus reservoirs and transmission routes [9].

Poliovirus WWS has been used for many years in several developed countries and in selected areas of developing countries [1]. In 2023, a total of 45 countries conducted poliovirus WWS at approximately 550 locations globally [9]. According to data from the EU-WISH questionnaire (a joint action project for Wastewater Integrated Surveillance for Public Health in Europe), 15 European Union (EU) countries had an established poliovirus WWS system in place before January 2024 [10]. However, depending on the national poliovirus epidemiological situation, the population vaccination coverage, and the objective of poliovirus WWS, countries face some notable challenges to optimise their national poliovirus WWS systems. These challenges include consideration of key characteristics of geographical areas (urbanisation level and settlement pattern), the catchment population (its size and location), the type and characteristics of sewage systems, laboratory capacity [11], and the selection of the most appropriate sampling method [12].

Two literature reviews have been conducted on the topic of the sampling schemes used for poliovirus WWS [11,12]. In these reviews, the authors have not only defined the advantages and disadvantages of individual sampling methods but also investigated the economic aspects and overall impact of poliovirus WWS, providing several important insights into sampling scheme design. Topics such as the selection of sampling sites, the determination of sampling frequency, and the choice of sample volume are also described in World Health Organisation (WHO) guidelines [8,9,13,14,15]. However, these WHO guidelines are written very generally and do not consider the direct application of guidelines in polio-free countries. The same applies to the above-mentioned literature reviews [11,12], where the differences between developed and developing countries were not fully addressed. In addition, the WWS objective, an essential prerequisite for the effective development of WWS system, was not explored. For this reason, this review aims to provide a summary of sampling schemes used for poliovirus WWS based on data from studies performed in EU countries, as well as analyse the sampling schemes in relation to the study objectives and compare data with studies from non-EU countries. Our review is important because understanding sampling schemes in developed countries and how they differ from those in developing countries supports the optimisation of poliovirus WWS systems in polio-free countries.

## 2. Materials and Methods

This literature review was conducted as part of the EU-WISH project with the objective of obtaining information on sampling schemes for poliovirus WWS. Based on the collected data, this review presents the main findings of the analysis related to sampling schemes described in the included studies and compares them between EU and non-EU countries. Despite this comparison, this analysis primarily focuses on data obtained from studies conducted in EU countries, as there are currently no available guidelines for optimising poliovirus WWS in polio-free countries.

### 2.1. Search and Selection of Relevant Studies

The selection of studies to identify the relevant studies for this literature review was based on three different sources, shown in the online Supplementary Materials, Figure S1. Firstly, we performed a literature search in the PubMed database using the search string: “polio” OR “poliovirus” OR “poliomyelitis” AND “wastewater surveillance” OR “environmental surveillance” OR “sewage surveillance”. The literature search covered studies published before 5 September 2024, with the search term being restricted to title/abstract search, observation period 2010–2024 and full-text studies. Studies were first screened by the study title, with the inclusion criterion being that the study addressed the topic of poliovirus WWS. In the next step, we screened the entire content of the studies and included studies that were in line with the aim of our literature review. Secondly, we included studies from the WHO document Guidelines on Environmental Surveillance for Detection of Polioviruses [8], which provides a review of global poliovirus detection in wastewater covering the period from 1991 to 2014. These were further screened, and those aligned with the aim of our literature review were included. Thirdly, we included additional relevant studies identified from a systematic review of the WWS of communicable diseases, conducted by Kilaru et al. [16], which covered literature published before 1 August 2021 from PubMed, SCOPUS, Science Direct and Google Scholar. To ensure data relevance, studies published before 1995 were excluded.

### 2.2. Data Analysis

The studies selected for the review were then extracted to obtain the data on the country of origin, WWS study objective, WWS study observation period, sampling site (e.g., wastewater treatment plant (WWTP), open canal, river), number of sampling sites, catchment population size of sampling sites, monitoring area of sample sites (geographic site), sampling frequency, sampling method (e.g., composite sample, grab sample, gauze pad) and sample volume collected. Data extraction additionally included information on laboratory methods for poliovirus detection and characterisation. The data obtained included information on the sampling schemes and the context of the studies.

During data processing, for the category WWS study objective, each study was classified into the respective group based on the study’s objective and context: establishment of WWS/new laboratory method; results of WWS; post-vaccination surveillance/oral polio vaccine (OPV) use; or response to a positive clinical or environmental event. A more detailed distinction between routine long-term surveillance and time-limited monitoring was not possible due to lack of this information in the publications. For the category sampling frequency, studies addressing response to poliovirus outbreak were classified according to the highest reported sampling frequency, reflecting adaptation to the study’s WWS objective. However, when studies reported different sampling frequencies for different sampling sites, these were assigned to multiple subcategories. Similarly, for the category wastewater sampling sites, studies were assigned into multiple subcategories if different sampling methods were used across sites. Consequently, in some categories, the number of observed variables may have exceeded the number of included studies, as individual studies were reported to multiple variables within the same category. For the category sample volume and category catchment population size, only studies reporting this information were included.

Our literature review analyses data from selected studies, including a comparison between studies conducted in EU and non-EU countries. To enable better comparisons, the number of studies has been converted into proportions, where the prevalence has been calculated separately for the studies from EU and non-EU countries, based on the total number of reported variables within each category. Further, a similar approach was applied for the analysis by WWS study objective, with excluding the variables “other” and “data not available”. It is important to bear in mind that the non-EU countries also include some developed countries, for example, countries from North and Central America.

## 3. Results

The selection of studies for the literature review included 1390 studies from the PubMed bibliographic database that met the search criteria and were published between 1 January 2010 and 5 September 2024. In addition, 24 studies from the WHO document [8] and 32 studies from the systematic literature review [16] were also included. Of these, 72 relevant studies were then selected for the qualitative analysis. The data obtained on the sampling schemes and the context of the studies, are shown in the online Supplementary Materials, Table S1.

Based on the country of origin, by continent, most studies were conducted in Europe (11 countries; 21 studies), followed by Asia (6 countries; 20 studies), Africa (9 countries; 16 studies), North and Central America (6 countries; 9 studies), Oceania (1 country; 4 studies) and South America (2 countries; 2 studies). The studies conducted in Europe included the following countries: Italy (six studies), the Netherlands (three studies), Greece (three studies), Estonia (one study), Finland (one study), Slovakia (two studies), France (one study), Poland (two studies), Cyprus (one study), and two non-EU countries, the United Kingdom (one study) and Russia (one study). Data on the WWS study objectives show that 68.4% (13 studies) studies from EU countries predominantly focused on “general WWS results”. EU studies also, but less frequently, focused on “establishment of WWS/new laboratory method” (three studies); “responding to a positive clinical case/positive wastewater sample” (two studies); and “post-vaccination surveillance/OPV use” (one study).

Most studies from EU and non-EU countries had conducted poliovirus WWS at WWTPs. Although representing a smaller proportion, studies from the EU also conducted WWS at sewage pipelines, airport sewage systems and essential poliovirus facilities (e.g., facilities used for vaccine production and poliovirus diagnostic laboratories) (Table 1). EU studies with the objective of “general WWS results” were predominantly carried out at WWTPs, whereas sampling in sewage pipelines was mainly associated with objectives “responding to a positive clinical case/positive wastewater sample” or “establishment of WWS/new laboratory method” (Table 2). Only studies from non-EU countries reported sampling wastewater from open canals and rivers (Table 1).

Most of the studies from the EU, and a large proportion of studies from non-EU countries, collected wastewater samples at locations with a catchment population between 100,000 and 300,000 population equivalents (PEs). In non-EU studies, the highest number of sampling sites covered catchment populations with fewer than 50,000 PE (Table 1). Nevertheless, in non-EU studies, WWS study objectives such as “general WWS results” and “responding to a positive clinical case/positive wastewater sample” were mostly associated with larger catchment populations, bigger than 100,000 PE (Table 2). The included data are shown in the online Supplementary Materials, Table S2.

In most EU and non-EU studies poliovirus WWS had been conducted on a monthly basis. However, the largest proportion of EU studies (38.1%) reported sampling between once a week and once a month (Table 1). In EU studies with the objective “general WWS results”, sampling was most often conducted once a month. More frequent than monthly sampling in EU studies was associated with the objectives “response to positive clinical or environmental event” and “establishment of WWS/new laboratory method”, as well as with “post-vaccination surveillance/OPV use” (Table 2).

In non-EU studies, the most commonly used sampling method was grab sampling (approximately 50%). In EU studies, the use of grab sampling and composite sampling was more evenly distributed, with both methods accounting for approximately 30% of the studies (Table 1). The same even distribution between grab and composite sampling was observed in EU studies with the objectives “general WWS results” and “establishing WWS/new laboratory method” (Table 2).

Analysis of grab sampling methods revealed that 8 out of 36 EU and non-EU studies provided additional details on sample collection. Among these, five non-EU studies indicated that sampling was conducted in the morning during peak flow, one non-EU study reported sampling in the afternoon between 14:00 and 15:00, and two non-EU studies mentioned sampling during an unspecified peak flow period. A similar analysis was conducted for composite sampling data, where 17 out of 18 EU and non-EU studies provided additional details on sample collection. Among them, 10 EU and non-EU studies reported the use of automatic samplers, while 7 EU and non-EU studies described combining grab samples. Automatic sampling was characterised by the continuous collection of wastewater over a full 24 h period using automatic samplers, which can do time-proportional (two studies) or flow-proportional (one study) sampling. In comparison to automatic samplers, composite samples created by combining grab samples generally covered a shorter duration, such as during 3 h peak flow periods or a 12 h collection period. In some cases, samples were taken at different times of the day or even on separate days (e.g., one grab sample in the morning and another in the afternoon, or samples combined from two different days).

**Figure 1:**
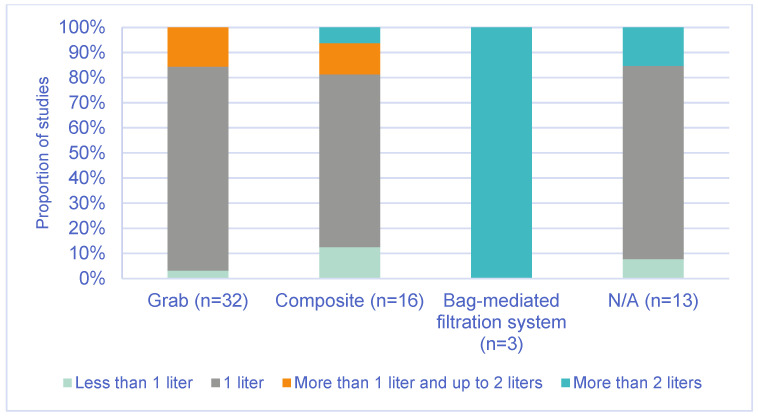
Studies included in the literature review from EU and non-EU countries by wastewater sample volume collected and wastewater sampling method (n = 64).

Most EU and non-EU studies (approximately 80%) collected 1 L of wastewater for laboratory analysis, regardless of the sampling method used (grab or composite). All studies that used bag filtration systems collected sample volumes greater than 2 L (Figure 1).

According to the primary laboratory detection methods described, there was little methodological divergence among the studies. Two of the 72 studies presented a general epidemiological overview and did not specify laboratory detection methods. Of the remaining 70 studies, only one (1.4%) employed direct real-time RT-PCR as the primary laboratory detection method. In the other 69 studies (98.6%), wastewater concentrates were inoculated into cell cultures according to the WHO protocol [4], after which additional methods were applied for viral typing or isolate characterisation.

Both cell lines recommended by the WHO protocol, RD and L20B, were used for laboratory detection. A poliovirus isolate was presumed when clear viral propagation was observed in the L20B cell line. Isolates were most commonly serotyped using a microneutralization assay (24 of 69 studies) or typed by molecular assays (28 of 69 studies) employing type- and variant-specific primers included in the molecular intratypic differentiation (ITD) kit. Thirteen studies did not report any typing method.

In 49 of the 70 studies, poliovirus isolates were further characterised by analysing mutation patterns through partial or complete sequencing of the VP1 coding region; four of these studies additionally included sequencing of the 5′ untranslated region (5′UTR). In two studies, 5′UTR sequencing alone was performed in combination with other ITD assays. Whole-genome sequencing was reported in four studies, one of which employed an amplicon-based Oxford Nanopore Technologies (ONT) approach.

## 4. Discussion

Sampling schemes for poliovirus WWS can be categorised into routine surveillance or enhanced surveillance, where enhanced surveillance can be implemented as a response to a positive poliovirus detection in wastewater or a confirmed clinical case. Our literature review showed that most studies from EU countries had the objective “general WWS results” [17,18,19,20,21,22,23,24,25,26,27,28,29], where, based on the reviewed study content, they most likely presented the results of national poliovirus WWS upon detection of cVDPV or WPV. Several studies also addressed topics such as “establishment of WWS/new laboratory method” [30,31,32], “responding to a positive clinical case/positive wastewater sample” [33,34] or “post-vaccination surveillance/OPV use” [35]. Less frequently, the WWS objectives in studies from EU countries were the feasibility of quantitative poliovirus WWS detection [30], WWS at an international airport [32] or response to an employee infection at an essential poliovirus facility [34]. Although Europe is a polio-free area, the continued presence of unvaccinated or under-vaccinated population groups, together with the fact that poliomyelitis has not yet been globally eradicated, means that the risk of reintroduction and spread of polioviruses in Europe persists [36]. To maintain polio-free status, WWS is therefore also important in Europe in order to confirm the absence of poliovirus circulation in the population [8].

Our literature review showed that in most studies from EU and non-EU countries, the sampling was conducted at WWTPs as the primary sampling site for poliovirus WWS [6,7,17,18,19,20,21,23,24,25,28,31,37,38,39,40,41,42,43,44,45,46,47,48,49,50,51,52,53,54,55,56,57,58,59,60,61,62,63,64,65,66]. The same was found in studies with the objective “general WWS results” [17,18,19,20,21,23,24,25,28]. Compared with non-EU studies [7,63,65,67,68,69,70,71], there was a higher proportion of EU studies [29,30,33] that conducted sampling in the sewer pipeline, although both had relatively low prevalence. Sampling in sewage pipelines was mostly used in EU studies with WWS study objectives “responding to a positive clinical case/positive wastewater sample” or “establishing WWS/new laboratory method”. In addition, EU studies have also reported sampling wastewater from airport sewage systems [32] and from essential poliovirus facilities [34]. Only studies from non-EU countries reported sampling wastewater from open canals [39,50,52,69,71,72,73,74] and rivers [45,54,65,66,75,76,77], which reflects the less-developed sewer infrastructure in some non-EU countries. Based on differences in sewer infrastructure reported in the reviewed studies, routine WWS in open canals and rivers is therefore less relevant for developed countries. GPEI guidelines [8] recommend selecting WWTPs as the primary sampling site, along with other larger wastewater collection points. This recommendation allows countries to decide independently, depending on the WWS objective and the availability of sampling options. Sampling in sewer networks may also offer advantages, as data on the catchment population and flow rate are often available, which is essential for further analysis and interpretation of WWS results.

Most EU studies [20,21,24,31], and a large proportion of non-EU studies [37,39,42,46,51,54,59,62,65,66,68,73], conducted poliovirus WWS at sampling sites that covered catchment populations between 100,000 and 300,000 population equivalents (PEs), which is consistent with GPEI recommendations [8]. In non-EU studies, sampling sites covering between 100,000 and 300,000 PEs were most commonly associated with the WWS study objectives “response to a positive clinical or environmental event” or “post-vaccination surveillance/OPV usage”, indicating the use of WWS in larger urban settings, where poliovirus may spread rapidly among susceptible populations due to characteristics of the urban environment [2]. Based on our literature review, the proportion of sampling sites covering more than 300,000 PE was similar between EU [21,24] and non-EU countries [37,38,39,44,48,50,51,62,65,68,71,73]. In larger catchment populations, a single wastewater sample can cover a greater number of people; however, the sensitivity of WWS is reduced. The lower sensitivity of the WWS system means that a higher number of infected individuals is required to detect poliovirus in a wastewater sample [8]. Most non-EU studies tend to use a lower catchment population below 50,000 PE [37,42,49,50,54,65,66,67,73], likely due to a more dispersed population and relatively smaller urban areas. In settings with smaller catchment populations and closely located sampling sites, a composite sample may be used by combining partial samples from different sampling locations to reduce the laboratory workload [8]. The entire national poliovirus WWS system in each EU country typically covers a population of between 300,000 and 7,000,000 PEs [10].

EU studies [18,19,21,22,24,28], together with non-EU studies [6,7,39,40,41,42,43,46,49,53,56,57,59,61,62,63,64,65,67,69,73,74,77,78,79,80,81], mostly sampled wastewater once a month, whereas most of the EU studies [20,21,23,24,25,31,34] reported sampling between once a week and once a month. Data from the EU-WISH questionnaire [10] show that sampling in EU countries is generally performed monthly or is adjusted as needed. This is consistent with WHO’s recommended sampling frequency of once per month [9,13]. However, countries not receiving direct GPEI funding may balance their sampling frequency based on their own risks of poliovirus circulation and the added value of poliovirus WWS compared with existing surveillance systems [9]. Analysis of sampling frequency by WWS study objective in EU countries showed that studies aiming to describe “general WWS results” [18,19,20,21,22,23,24,25,28] generally conducted sampling once per month or even less frequently. In contrast, EU studies focused on “establishment of WWS/new laboratory method” [30,32] or “post-vaccination surveillance/OPV use” [35] applied weekly or more frequent sampling. When interpreting these results, especially for EU studies, it is important to consider that higher sampling frequencies (more than once per month) may also reflect a response to a poliovirus outbreak or the detection of a positive wastewater sample. According to the questionnaire of the European Centre for Disease Prevention and Control from January 2025, only Lithuania, Ireland, Italy, Poland, Romania, Finland and Germany (for some sampling sites) reported routine poliovirus WWS with a sampling frequency higher than once per month [82]. Higher sampling frequency than once per month is recommended by WHO in situations where poliovirus has been detected in the country [9,13]. Accordingly, non-EU studies with the objective “general WWS results” tended to report higher sampling frequencies than EU studies.

Our literature review indicates that approximately 50% of non-EU studies [37,39,43,44,45,47,50,51,52,53,57,58,59,60,61,62,63,67,68,69,71,73,74,77,78,80,81,83,84,85] used grab sampling for WWS. In EU studies, the use of grab [18,19,28,29,32,33] and composite sampling [20,21,23,25,31] was more evenly balanced, with each method accounting for around 30% of all studies. However, a high proportion of EU studies did not specify the sampling method [17,22,24,26,27,30,34,35], and the actual distribution of sampling methods in EU countries therefore remains uncertain. Composite samples are more representative than grab samples, as they consist of multiple samples collected over a longer time period, thereby capturing a larger portion of the catchment population. WHO currently recommends grab sampling for poliovirus WWS, while 24 h composite sampling is defined as the ideal sampling method because it provides the most representative data [9]. Differences in the implementation of grab and composite sampling, regardless of country of origin are difficult to interpret, as they depend on several factors, including the characteristics of the sampling site, financial resources, and other logistical factors. Among EU and non-EU studies that used grab sampling, most reported sampling in the morning during peak wastewater flow [39,52,71,78,83], while studies that used composite sampling most commonly reported the use of automatic samplers [7,20,21,31,37,40,41,42,53,56]. The gauze method (trap method) was rarely used in EU [28] and non-EU studies [40,55], probably due to logistical difficulties and lower sensitivity [2]. According to GPEI recommendations, the gauze method is not the preferred method for WWS because it is difficult to ensure adequate standardisation of absorbent material [8]. Furthermore, long-term experience shows that WWS systems that use concentrated grab samples more frequently detect polioviruses and non-polio enteroviruses than those using trap samples [2]. The bag-mediated filtration method has been used only experimentally in a few studies from non-EU countries [50,68,69,85], while the WHO recommends this method solely as an alternative method [9].

About 80% of EU [17,18,19,20,21,23,28,29,31,33,35,83] and non-EU studies [7,39,44,45,46,47,48,49,50,53,54,57,59,60,61,63,64,66,67,68,69,70,72,73,74,75,77,79,80,81,83,84,85,86], regardless of the sampling method (grab or composite sampling), sampled 1 L of wastewater for laboratory analysis, which is consistent with WHO [9] and GPEI [8] recommendations. For the bag-mediated filtration method, larger sample volumes are generally required, typically between 5 and 10 L of wastewater [9].

For the laboratory detection method and characterisation of poliovirus in wastewater samples, the WHO protocol [4] remains the most widely used approach and represents the only fully standardised analytical process. However, with the development of new molecular techniques, it is now possible to re-evaluate the current strategy and assess the added value of novel methods, such as first-line detection using digital PCR (dPCR) or real-time RT-PCR. These approaches are particularly appropriate for poliovirus-free countries, where rapid detection is essential for timely assessment of potential virus introduction into the population under surveillance. Nevertheless, virus isolation must remain the key indicator of a genuine public health risk in environmental samples, as it provides confirmation of the presence of infectious virus.

Despite the relatively small number of studies from the EU countries, our study provides insight into the sampling schemes used in polio-free countries. The results indicate that most EU countries conduct poliovirus WWS directly in line with WHO recommendations [8,9,13,14,15], despite these being primarily designed for developing countries with a high risk of poliovirus circulation. Based on this, optimisation of existing WWS systems would be beneficial to ensure their long-term sustainability. One of the key gaps identified in current guidelines for poliovirus WWS is that there is no recommended sampling frequency for polio-free countries. Nevertheless, guidance proposes that countries balance their sampling frequency based on their specific risk of poliovirus introduction and circulation, as well as the added value of poliovirus WWS compared with existing surveillance systems for polio. Another gap identified in EU countries relates to the choice of sampling method, where the use of 24 h composite sampling should be recommended, as it provides the most representative data and is more suitable for detecting potential poliovirus introduction into polio-free country. The design of WWS systems should be implemented according to WWS objectives and how the collected data will be used for public health measures. Overall, our findings highlight the need for guidelines that reflect the epidemiological context of polio-free countries and support optimisation of national WWS systems.

### 4.1. Strengths and Limitations

Our literature review provides a structured overview of sampling schemes for poliovirus WWS, covering studies from three different sources: the PubMed database, a WHO literature review, and a systematic literature review by Kilaru et al. [16] on communicable disease WWS, which included a search for studies from PubMed, Scopus, ScienceDirect and Google Scholar. We believe that by including all those sources, we ensured good coverage of relevant literature and minimised the possibility of missing key studies. In addition, the comparison of the included studies with the WHO and GPEI guidelines provides additional value.

The main limitation of our literature review is the low number of studies from EU countries, which constrains the ability to draw firm conclusions about sampling schemes in Europe. In addition, it is important to bear in mind that the included studies do not necessarily reflect complete national poliovirus WWS systems. A further limitation is that some studies conducted in developed countries (e.g., United Kingdom, the United States of America) were categorised as non-EU studies and therefore, the non-EU category does not necessarily fully correspond to developing countries. Nevertheless, our literature review presents the most relevant sampling schemes currently used in EU and non-EU countries. The findings of the EU-WISH questionnaire [10], which provides data on national poliovirus WWS systems in EU countries, support the conclusions of our literature review in comparable categories.

In this literature review, we have only presented sampling methods described in the included literature. The financial considerations of individual methods were not analysed, as the objective of our review was to provide a general overview of the sampling schemes used in both EU and non-EU countries. Nevertheless, the data presented may support countries in selecting appropriate sampling schemes, as it provides insight into existing practices.

### 4.2. Current Research Gaps

Several studies have focused on microbiological analysis of polioviruses in wastewater, describing either limited-time monitoring (i.e., 1 year or less) conducted in “response to a positive clinical case/positive wastewater sample” [6,7,34,37,39,72], “establishment of WWS/new laboratory method” [30,42,69,71,78,81,84] or “post-vaccination surveillance/OPV use” [45,49,70,86], while other studies have addressed long-term poliovirus WWS [24,25,26,28,29,38,40,41,47,53,57,58,63,75,87,88]. In recent years, the number of studies from developing countries has increased [43,45,47,50,52,58,59,61,63,64,67,68,69,70,74,77,78,79,80,81,83,84,85,87], due to GPEI’s efforts to achieve the global poliovirus eradication. Regardless, studies generally do not describe the national poliovirus WWS systems in detail.

Current research shows an important gap in the availability of detailed information on the background behind the design of national sampling schemes and the adaptation of WWS systems to specific poliovirus situations. This topic was partially addressed in our literature review and in the systematic literature review conducted by Duintjer Tebbens et al. [11]. In addition, the public health relevance of detecting poliovirus in wastewater in the absence of clinical cases and associated public health measures in such situations remains understudied.

### 4.3. Further Research

To further deepen knowledge about poliovirus WWS, it would be valuable to collect detailed information on the sampling schemes used in individual countries, both developed and developing, and to clarify the rationale and decision-making behind the implementation of these WWS systems. It is also important to assess the effectiveness of existing WWS systems in relation to the current global and national poliovirus epidemiological situation. The exchange of good practices between countries plays a key role in the establishment of an effective and cost-justified WWS system. These activities are already ongoing among EU countries under the EU-WISH project and have been further expanded to non-EU countries through the Global Consortium for Wastewater and Environmental Surveillance for Public Health (GLOWACON).

## 5. Conclusions

This literature review provides a summary of the sampling schemes used in EU and non-EU studies for poliovirus WWS. The literature review results show that most countries conduct poliovirus WWS in line with WHO recommendations, within the limits of their capabilities. However, these guidelines are primarily designed for developing countries with a higher risk of poliovirus circulation, highlighting the need for adaptation in polio-free countries. In this context, EU countries may consider optimising their systems by adapting sampling schemes to their own risks of poliovirus introduction and circulation and the added value of poliovirus WWS compared with existing surveillance systems. Further, the results show important differences between sampling schemes in EU and non-EU countries, where differences in financial resources, existing infrastructure and laboratory capacity lead to variability in the implementation of WWS systems. This highlights the need for a systematic comparison of countries’ practices and experiences, as well as for guidelines that reflect the epidemiological context of polio-free countries, which can significantly contribute to the further optimisation of WWS systems and to the more effective use of available resources.

## Figures and Tables

**Table 1:** Comparison of different sampling schemes for poliovirus wastewater surveillance in studies from European Union and non-European Union countries (n = 72).

Sampling Scheme Characteristics		EU Countries		Non-EU Countries	
Category	Description of Variables	N	Prevalence [%]	N	Prevalence [%]
**Wastewater** **sampling sites (n = 72)**	WWTP	10	52.6	32	45.7
	Open canals	0	0.0	8	11.43
	Sewage pipelines	3	15.8	8	11.43
	River water	0	0.0	7	10.0
	N/A	4	21.1	10	14.29
	Other	2	10.5	5	7.14
**The PE covered with wastewater sampling sites (n = 25)**	<50,000	4	8.5	53	44.2
	50,000–99,999 PE	6	12.8	11	9.2
	100,000–300,000 PE	26	55.3	32	26.7
	>300,000 PE	11	23.4	24	20.0
**Frequency of wastewater sampling (n = 72)**	Once a week or more	3	14.3	12	21.8
	Between once a week and once a month	8	38.1	78	14.5
	Once a month	6	28.6	27	49.1
	Less than once a month	1	4.8	0	0
	N/A	3	14.3	8	14.6
**Wastewater sampling methods used for** **poliovirus wastewater surveillance (n = 72)**	Grab	6	30.0	30	50.0
	Composite	5	25.0	10	16.7
	Gauze pad	1	5.0	2	3.3
	Bag-mediated filtration system	0	0.0	5	8.3
	N/A	8	40.0	13	21.7

EU—European Union; n—number of studies; N—number of studies for each variable; N/A—data not available; PE—population equivalent.

**Table 2:** Comparison of different sampling schemes for poliovirus wastewater surveillance based on study wastewater surveillance objective in studies from European Union and non-European Union countries.

	EU Countries				Non-EU Countries			
Study Objective of Monitoring	Wastewater Sampling Sites (n = 13)				Wastewater Sampling Sites (n = 40)			
	WWTP	Open Canals	Sewage Pipelines	River Water	WWTP	Open Canals	Sewage Pipelines	River Water
**Establishing wastewater** **surveillance/new** **laboratory method**	50.0%	0.0%	50.0%	0.0%	27.3%	36.3%	27.3%	9.1%
**Results of wastewater surveillance**	90.0%	0.0%	10.0%	0.0%	70.0%	5.0%	15.0%	10.0%
**Post-vaccination** **surveillance/OPV** **usage**	0.0%	0.0%	0.0%	0.0%	54.5%	0.0%	9.1%	36.4%
**Response to positive clinical or** **environmental event**	0.0%	0.0%	100.0%	0.0%	60.0%	30.0%	10.0%	0.0%
	**The PE Covered with Wastewater Sampling Sites (n = 5)**				**The PE Covered with Wastewater Sampling Sites** **(n = 17)**			
	<50,000 PE	50,000–99,999 PE	100,000–300,000 PE	>300,000 PE	<50,000 PE	50,000–99,999 PE	100,000–300,000 PE	>300,000 PE
**Establishing wastewater** **surveillance/new** **laboratory method**	25.0%	0.0%	75.0%	0.0%	63.2%	15.8%	5.3%	15.8%
**Results of wastewater surveillance**	7.0%	14.0%	53.5%	25.6%	35.7%	0.0%	28.6%	35.7%
**Post-vaccination** **surveillance/OPV** **usage**	0.0%	0.0%	0.0%	0.0%	60.0%	0.0%	36.0%	4.0%
**Response to positive clinical or** **environmental event**	0.0%	0.0%	0.0%	0.0%	15.4%	19.2%	46.2%	19.2%
	**EU Countries**				**Non-EU Countries**			
**Study objective of** **monitoring**	**Frequency of Wastewater Sampling (n = 16)**				**Frequency of Wastewater Sampling (n = 43)**			
	Once a week or more	Between once a week and once a month	Once a month	Less than once a month	Once a week or more	Between once a week and once a month	Once a month	Less than once a month
**Establishing wastewater** **surveillance/new** **laboratory method**	66.7%	33.3%	0.0%	0.0%	23.1%	7.7%	69.2%	0.0%
**Results of wastewater surveillance**	0.0%	41.7%	50.0%	8.3%	21.4%	28.6%	50.0%	0.0%
**Post-vaccination** **surveillance/OPV** **usage**	100.0%	0.0%	0.0%	0.0%	55.6%	11.1%	33.3%	0.0%
**Response to positive clinical or** **environmental event**	0.0%	100.0%	0.0%	0.0%	11.1%	22.2%	66.7%	0.0%
	**Wastewater Sampling Methods Used For** **Poliovirus Wastewater Surveillance (n = 11)**				**Wastewater Sampling Methods Used For** **Poliovirus Wastewater Surveillance (n = 37)**			
	Grab	Composite	Gauze pad	Bag-mediated filtration system	Grab	Composite	Gauze pad	Bag-mediated filtration system
**Establishing wastewater** **surveillance/new** **laboratory method**	50.0%	50.0%	0.0%	0.0%	73.3%	13.3%	0.0%	13.3%
**Results of wastewater surveillance**	44.4%	44.4%	11.1%	0.0%	56.3%	18.8%	12.5%	12.5%
**Post-vaccination** **surveillance/OPV** **usage**	0.0%	0.0%	0.0%	0.0%	57.1%	28.6%	0.0%	14.3%
**Response to positive clinical or** **environmental event**	100.0%	0.0%	0.0%	0.0%	57.1%	42.9%	0.0%	0.0%

EU—European Union; n—number of studies; PE—population equivalent.

## Data Availability

The original contributions presented in this study are included in the article/Supplementary Material. Further inquiries can be directed to the corresponding author.

## Supplementary Materials

The following supporting information can be downloaded at: https://www.mdpi.com/article/10.3390/microorganisms14040861/s1, Figure S1: A flow chart of the selection of studies for the literature review; Table S1: Extracted data from studies of literature review of sampling schemes; Table S2: Additional extracted data from studies of the literature review of catchment populations covered by sampling sites.
