# Supplementary material for: Sampling Schemes in Poliovirus Wastewater Surveillance Studies from European Countries and Their Comparison to Other Studies: A Literature Review
Source: Microorganisms. 2026 Apr 11;14(4):861. doi: 10.3390/microorganisms14040861 (PMC13119512; doi:10.3390/microorganisms14040861)
Supplement: Supplementary file 1 [file microorganisms-14-00861-s001.zip › Supplement Table S2.pdf]

Table S2. Additional extracted data from studies of the literature review on catchment population covered by sampling sites.

Country	Number	Monitoring area	Sampling site	Population equivalent covered
Egypt [1]	1	Aswan City	WWTP	238,120
	2	Asyut City	WWTP	375,762
	3	Asyut/Dairut	Open canals	60,660
	4	Beni-Suef	WWTP	188,633
	5	Fayoum City	WWTP	285,050
	6	Minya/Abo Qurqas	WWTP	54,731
	7	Minya City	WWTP	220,563
	8	Minya/Mallawy	Open canals	130,614
	9	Qena City	WWTP	160,917
	10	Sohang City	WWTP	185,577
	11	Tanta City	WWTP	397,668
India [2]	12	Wadala sewage pumping station	Sewage pumping station	400,000
	13	Dharavi slum	Large trenches draining domestic wastewater	590,000
	14	Shivajinagar slum	Large trenches draining domestic wastewater	762,000
Russia [3]	15	Moscow treatment plant 1	WWTP	6,000,000
	16	Moscow treatment plant 2	WWTP	4,000,000
	17	Moscow treatment plant 3	WWTP	1,000,000
	18	Moscow treatment plant 4	WWTP	1,000,000
Pakistan [4]	19	Kilah Adbullah	Trenches containing sewage	2,893
	20	DIKhan	Trenches containing sewage	11,403
	21	Peshawar	Trenches containing sewage	9,653
	22	Islamabad	Trenches containing sewage	7,682
	23	Rawalpindi	Trenches containing sewage	21,526
	24	Lahore	Pumping station	20,475
	25	Faisalabad	Pumping station	26,871
	26	Mutan	Trenches containing sewage	19,190
	27	Sukkur	Trenches containing sewage	7,415
	28	Hyderabad	Pumping station	7,474
	29	Baldia	Trenches containing sewage	2,919

	30	Gadap	Trenches containing sewage	8,062
	31	Gulshan Iqbal (Karachi)	Trenches containing sewage	6,151
	32	Quetta	Trenches containing sewage	4,640
Israel [**5**]	33	Tel Sheva	WWTP	17,500
	34	Jaljulia	WWTP	9,100
	35	Kselfe	WWTP	18,000
	36	Lod	WWTP	72,000
	37	Ramle	WWTP	69,000
	38	Beer-Sheva	WWTP	199,000
	39	Rahat	WWTP	59,000
India [**6**]	40	N/A	WWTP	1,000,000
Netherlands [**7**]	41	N/A	Pumping station	37,000
Japan [**8**]	42	Kyushu Plant T	WWTP	190,000
	43	Kyushu Plant Y	WWTP	180,000
Italy [**9**]	44	Bolzano	WWTP	374,000
	45	Milan (Nosedo)	WWTP	300,000
	46	Milan (Nosedo Est)	WWTP	300,000
	47	Milan (Peschiera)	WWTP	300,000
	48	Venice (Cavarzere)	WWTP	17,500
	49	Venice (Ceggia)	WWTP	5,000
	50	Venice (Musile)	WWTP	10,000
	51	Venice (Campalto)	WWTP	110,000
	52	Venice (Fusina)	WWTP	330,000
	53	Parma (Est)	WWTP	130,000
	54	Parma (Ovest)	WWTP	160,000
	55	Sassari (Caniggia)	WWTP	120,000
	56	Bari (Fesca)	WWTP	300,000
	57	Bari (Mola)	WWTP	300,000
	58	Bari (Japigia)	WWTP	300,000
	59	Naples (Cuma)	WWTP	1,000,000
	60	Naples (S.G. Teduccio)	WWTP	700,000
	61	Naples (Est)	WWTP	500,000
	62	Palermo (Acqua dei Corsari)	WWTP	130,000
	63	Palermo (Fondo Verde)	WWTP	70,000
	64	Palermo (Via Diaz)	WWTP	70,000
	65	Palermo (Jolly Hotel)	WWTP	70,000
Brazil [**10**]	66	Rio de Janeiro	WWTP	1,500,000
Colombia [**11**]	67	Quimbaya (Aldana)	WWTP	46,614
	68	El Cafetero (C. Diablo)	WWTP	37,808
	69	Rufino Jose, Cuervo Sur (Sta Rita)	WWTP	49,224
	70	Centenario (Pinares)	WWTP	33,111
	71	Rufino Jose, Cuervo Sur (Cristales)	WWTP	49,224
	72	Francisco de, Paula Santander (Miraflores)	WWTP	14,429
	73	Alfonso Lopez (M Beltran)	WWTP	33,796

	74	Rufino Jose, Cuervo Sur (Los Quindos)	WWTP	49,224
Haiti [12]	75	Canal Saint Georges	Open canal	397,494
	76	Bois de Chene	Open canal	216,089
	77	Key Soleil Bridge	Open canal	47,091
	78	Key Soleil Health Facility	Open canal	25,703
India [13]	79	Lucknow city Plant 1	WWTP	1,000,000
	80	Lucknow city Plant 2	WWTP	600,000
	81	Lucknow city Plant 3	WWTP	450,000
	82	Lucknow city Plant 4	WWTP	150,000
South Africa [14]	83	Daspoort	WWTP	200,000
	84	Daveyton	WWTP	13,000
	85	Grundlingh	WWTP	5,000
	86	Heidelberg	WWTP	10,000
	87	Mccomb	WWTP	5,000
	88	Modderfontein	WWTP	3,000
	89	Olifantsfontein	WWTP	100,000
	90	Tsakane	WWTP	20,000
	91	Rynfield	WWTP	20,000
	92	Vlakplaats	WWTP	130,000
Italy [15]	93	Peschiera Borromeo	WWTP	270,000
	94	Nosedo	WWTP	300,000
	95	Nosedo Est	WWTP	300,000
Italy [16]	96	Parma (West plant)	WWTP	168,000
	97	Parma (East plant)	WWTP	180,000
England [17]	98	Costons Lane Northolt Lane (Ealing)	WWTP	48,211
	99	Richford Street (Ealing)	WWTP	52,936
	100	Waterside Close Hendon (Wembley)	WWTP	209,706
	101	Watkin Road Wembley (Wembley)	WWTP	185,957
	102	Rayners Lane Harrow (Harrow)	WWTP	25,735
	103	Martaban Road (Islington)	WWTP	302,946
	104	Gascoigne Road (Redbridge)	WWTP	364,674
	105	Effra Branch Sewer (Lambeth)	WWTP	58,150
	106	Folkstone Road (Newham)	WWTP	253,476
	107	Fraser Road (Bexley)	WWTP	25,935
	108	West Ham Inlet (Newham)	WWTP	184,069
	109	Isle of Dogs (Tower Hamlets)	WWTP	95,590
	110	Cadogan Terrace (Waltham Forest)	WWTP	289,321
	111	Brookmill Road (Lewisham)	WWTP	21,499
	112	Greenwich Terminal (Wandsworth)	WWTP	886,210
Haïti [18]	113	Port au Prince (Bois de Neuf)	Open canal	347,237
	114	Port au Prince (Bois de Chene)	Open canal	339,624
	115	Port au Prince (Route Rails Diquini)	Open canal	67,320
	116	Port au Prince (Cite au Cayes)	Open canal	139,987
	117	Port au Prince (Carrefour)	Open canal	9,361
	118	Gonaïves (Key Soleil Health Facility)	Open canal	20,749
	119	Gonaïves (Key Soleil Bridge)	Open canal	82,123
	120	Gonaïves (Avenue Leon Legros)	Open canal	10,034

	121	Gonaïves (Boulevard de I' Avenir)	Open canal	20,241
	122	Gonaïves (Key Soleil School Saint Marc)	Open canal	17,878
	123	Saint Marc (Avenue Maurepas)	Open canal	50,744
	124	Saint Marc (Impasse Hucar)	Open canal	5,655
	125	Saint Marc (Rue Petion Cap Haitien)	Open canal	49,372
Italy [19]	126	Milan (Milan Nosedo)	WWTP	300,000
	127	Milan (Milan Nosedo Est)	WWTP	300,000
	128	Milan (Milan Peschiera)	WWTP	300,000
	129	Parma (Parma Est)	WWTP	130,000
	130	Parma (Parma Ovest)	WWTP	160,000
	131	Rome (Rome Nord)	WWTP	490,000
	132	Rome (Rome Est)	WWTP	720,000
	133	Rome (Rome Sud)	WWTP	1,000,000
	134	Bari (Bari Fesca)	WWTP	300,000
	135	Bari (Mola di Bari)	WWTP	300,000
	136	Bari (Bari Japigia)	WWTP	300,000
	137	Palermo (Acqua dei Corsari)	WWTP	130,000
	138	Palermo (Fondo Verde)	WWTP	70,000
	139	Palermo (Via Diaz)	WWTP	70,000
	140	Palermo (Jolly Hotel)	WWTP	70,000
	141	Naples (Naples Cuma)	WWTP	1,000,000
	142	Naples (Naples Teduccio)	WWTP	700,000
	143	Naples (Naples Est)	WWTP	500,000
	144	Sassari (Sassari)	WWTP	120,000
Panama (USA) [20]	145	Nuevo Tocumen (Panama City)	WWTP	115,151
	146	Las Mendozas, Villa Real (La Chorrera)	WWTP	165,000
	147	David, Las Lomas (Chiriqui)	WWTP	144,858
Mexico [21]	148	Hidalgo site	Access points to underground wastewater canals	270,000
	149	Mexico City site	Access points to underground wastewater canals	4,000,000
Japan [22]	150	WTP-A	WWTP	200,000
	151	WWTP-B	WWTP	500,000
Israel [23]	152	Sorek	Basin and WWTP	680,000
	153	Jerusalem-Og	WWTP	220,000
	154	Jerusalem-Har Homa	WWTP	38,000
	155	Jerusalem-Kldron river	River	300,000
	156	Bet Shemesh area	City manhole and WWTP	225,000
	157	Bnel Brak area	City manholes	220,000
South Africa [24]	158	Daspoort	WWTP	200,000
	159	Daveyton	WWTP	13,000
	160	Grundlingh	WWTP	5,000
	161	Heidelberg	WWTP	10,000

	162	Mccomb	WWTP	5,000
	163	Modderfontein	WWTP	3,000
	164	Olifantsfontein	WWTP	100,000
	165	Tskane	WWTP	20,000
	166	Rybfuekd	WWTP	20,000
	167	Vlakplaats	WWTP	130,000

WWTP – Wastewater Treatment Plant; N/A – Data Not Available.

References

[1] El Bassioni, L; Barakat, I; Nasr, E; de Gourville, E. M.; Hovi, T.; Blomqvist, S.; Burns, C.; Stenvik, M.; Gary, H.; Kew, O. M.; et al. Prolonged Detection of Indigenous Wild Polioviruses in Sewage from Communities in Egypt. *Am. J. Epidemiol.* **2003**, *158 (8)*, 807–815, https://doi.org/10.1093/aje/kwg202.

[2] Deshpande, J. M.; Shetty, S. J.; Siddiqui, Z. A. Environmental Surveillance System to Track Wild Poliovirus Transmission. *Appl. Environ. Microbiol.* **2003**, *69 (5)*, 2919–2927, https://doi.org/10.1128/AEM.69.5.2919-2927.2003.

[3] Ivanova, O. E.; Yarmolskaya, M. S.; Eremeeva, T. P.; Babkina, G. M.; Baykova, O. Y.; Akhmadishina, L. V.; Krasota, A. Y.; Kozlovskaya, L. I.; Lukashev, A. N. Environmental Surveillance for Poliovirus and Other Enteroviruses: Long-Term Experience in Moscow, Russian Federation, 2004–2017. *Viruses* **2019**, *11 (5)*, 424, https://doi.org/10.3390/v11050424.

[4] O'Reilly, K. M.; Verity, R.; Durry, E.; Asghar, H.; Sharif, S.; Zaidi, S. Z.; Wadood, M. Z. M.; Diop, O. M.; Okayasu, H.; Safdar, R. M.; et al. Population Sensitivity of Acute Flaccid Paralysis and Environmental Surveillance for Serotype 1 Poliovirus in Pakistan: An Observational Study. *BMC Infect. Dis.* **2018**, *18 (1)*, 176, https://doi.org/10.1186/s12879-018-3070-4.

[5] Berchenko, Y.; Manor, Y.; Freedman, L. S.; Kaliner, E.; Grotto, I.; Mendelson, E.; Huppert, A. Estimation of Polio Infection Prevalence from Environmental Surveillance Data. *Sci. Transl. Med.* **2017**, *9 (383)*, eaaf6786, https://doi.org/10.1126/scitranslmed.aaf6786.

[6] Tiwari, S.; Dhole, T. N. Assessment of Enteroviruses from Sewage Water and Clinical Samples during Eradication Phase of Polio in North India. *Virol. J.* **2018**, *15 (1)*, 157, https://doi.org/10.1186/s12985-018-1075-7.

[7] Lodder, W. J.; Buisman, A. M.; Rutjes, S. A.; Heijne, J. C.; Teunis, P. F.; de Roda Husman, A. M. Feasibility of Quantitative Environmental Surveillance in Poliovirus Eradication Strategies. *Appl. Environ. Microbiol.* **2012**, *78 (11)*, 3800–3805, https://doi.org/10.1128/AEM.07972-11.

[8] Nakamura, T.; Hamasaki, M.; Yoshitomi, H.; Ishibashi, T.; Yoshiyama, C.; Maeda, E.; Sera, N.; Yoshida, H. Environmental Surveillance of Poliovirus in Sewage Water around the Introduction Period for Inactivated Polio Vaccine in Japan. *Appl. Environ. Microbiol.* **2015**, *81 (5)*, 1859–1864, https://doi.org/10.1128/AEM.03575-14.

[9] Delogu, R.; Battistone, A.; Buttinelli, G.; Fiore, S.; Fontana, S.; Amato, C.; Cristiano, K.; Gamper, S.; Simeoni, J.; Frate, R.; et al. Poliovirus and Other Enteroviruses from Environmental Surveillance in Italy, 2009-2015. *Food Environ. Virol.* **2018**, *10 (4)*, 333–342, https://doi.org/10.1007/s12560-018-9350-8.

[10] de Oliveira Pereira, J. S.; da Silva, L. R.; de Meireles Nunes, A.; de Souza Oliveira, S.; da Costa, E. V.; da Silva, E. E. Environmental Surveillance of Polioviruses in Rio de Janeiro, Brazil, in Support to the Activities of Global Polio Eradication Initiative. *Food Environ. Virol.* **2016**, *8 (1)*, 27–33, https://doi.org/10.1007/s12560-015-9221-5.

[11] González, M. M.; Fonseca, M. C.; Rodríguez, C. A.; Giraldo, A. M.; Vila, J. J.; Castaño, J. C.; Padilla, L.; Sarmiento, L. Environmental Surveillance of Polioviruses in Armenia, Colombia before Trivalent Oral Polio Vaccine Cessation. *Viruses* **2019**, *11 (9)*, 775, https://doi.org/10.3390/v11090775.

[12] Coulliette-Salmond, A. D.; Alleman, M. M.; Wilnique, P.; Rey-Benito, G.; Wright, H. B.; Hecker, J. W.; Miles, S.; Peñaranda, S.; Lafontant, D.; Corvil, S.; et al. Haiti Poliovirus Environmental Surveillance. *Am. J. Trop. Med. Hyg.* **2019**, *101 (6)*, 1240–1248, https://doi.org/10.4269/ajtmh.19-0469.

[13] Chowdhary, R.; Dhole, T. N. Interrupting Wild Poliovirus Transmission Using Oral Poliovirus Vaccine: Environmental Surveillance in High-Risks Area of India. *J. Med. Virol.* **2008**, *80 (8)*, 1477–1488, https://doi.org/10.1002/jmv.21230.

[14] Pavlov, D. N. Poliovirus Vaccine Strains in Sewage and River Water in South Africa. *Can. J. Microbiol.* **2006**, *52 (8)*, 717–723, https://doi.org/10.1139/w06-026.

[15] Pellegrinelli, L.; Binda, S.; Chiaramonte, I.; Primache, V.; Fiore, L.; Battistone, A.; Fiore, S.; Gambino, M.; Bubba, L.; Barbi, M. Detection and Distribution of Culturable Human Enteroviruses through Environmental Surveillance in Milan, Italy. *J. Appl. Microbiol.* **2013**, *115 (5)*, 1231–1239, https://doi.org/10.1111/jam.12321.

[16] Cesari, C.; Colucci, M. E.; Veronesi, L.; Giordano, R.; Paganuzzi, F.; Affanni, P.; Bracchi, M. T.; Capobianco, E.; Ferrari, G.; Tanzi, M. L. Detection of Enteroviruses from Urban Sewage in Parma. *Acta Biomed.* **2010**, *81 (1)*, 40–46.

[17] Klapsa, D.; Wilton, T.; Zealand, A.; Bujaki, E.; Saxentoff, E.; Troman, C.; Shaw, A. G.; Tedcastle, A.; Majumdar, M.; Mate, R.; et al. Sustained Detection of Type 2 Poliovirus in London Sewage between February and July, 2022, by Enhanced Environmental Surveillance. *Lancet.* **2022**, *400 (10362)*, 1531–1538, https://doi.org/10.1016/S0140-6736(22)01804-9.

[18] Alleman, M. M.; Coulliette-Salmond, A. D.; Wilnique, P.; Belgasmi-Wright, H.; Sayyad, L.; Wong, K.; Gue, E.; Barrais, R.; Rey-Benito, G.; Burns, C. C.; et al. Environmental Surveillance for Polioviruses in Haïti (2017-2019): The Dynamic Process for the Establishment and Monitoring of Sampling Sites. *Viruses* **2021**, *13 (3)*, 505, https://doi.org/10.3390/v13030505.

[19] Battistone, A.; Buttinelli, G.; Fiore, S.; Amato, C.; Bonomo, P.; Patti, A. M.; Vulcano, A.; Barbi, M.; Binda, S.; Pellegrinelli, L.; et al. Sporadic Isolation of Sabin-like Polioviruses and High-Level Detection of Non-Polio Enteroviruses during Sewage Surveillance in Seven Italian Cities, after Several Years of Inactivated Poliovirus Vaccination. *Appl. Environ. Microbiol.* **2014**, *80 (15)*, 4491–4501, https://doi.org/10.1128/AEM.00108-14.

[20] Rojas-Bonilla, M.; Coulliette-Salmond, A.; Belgasmi, H.; Wong, K.; Sayyad, L.; Vega, E.; Grimoldi, F.; Oberste, M. S.; Rüttimann, R. Environmental Surveillance for Risk Assessment in the Context of a Phase 2 Clinical Trial of Type 2 Novel Oral Polio Vaccine in Panama. *Viruses* **2021**, *13 (7)*, 1355, https://doi.org/10.3390/v13071355.

[21] Estívariz, C. F.; Pérez-Sánchez, E. E.; Bahena, A.; Burns, C. C.; Gary, H. E., Jr.; García-Lozano, H.; Rey-Benito, G.; Peñaranda, S.; Castillo-Montufar, K. V.; Nava-Acosta, R. S.; et al. Field Performance of Two Methods for Detection of Poliovirus in Wastewater Samples, Mexico 2016-2017. *Food Environ. Virol.* **2019**, *11 (4)*, 364–373, https://doi.org/10.1007/s12560-019-09399-9.

[22] Kitakawa, K.; Kitamura, K.; Yoshida, H. Monitoring Enteroviruses and SARS-CoV-2 in Wastewater Using the Polio Environmental Surveillance System in Japan. *Appl. Environ. Microbiol.* **2023**, *89 (4)*, e0185322, https://doi.org/10.1128/aem.01853-22.

[23] Zuckerman, N. S.; Bar-Or, I.; Sofer, D.; Bucris, E.; Morad, H.; Shulman, L. M.; Levi, N.; Weiss, L.; Aguvaev, I.; Cohen, Z.; et al. Emergence of Genetically Linked Vaccine-Originated Poliovirus Type 2 in the Absence of Oral Polio Vaccine, Jerusalem, April to July 2022. *Euro Surveill.* **2022**, *27 (37)*, 2200694, https://doi.org/10.2807/1560-7917.ES.2022.27.37.2200694.

[24] Pavlov, D. N.; Van Zyl, W. B.; Van Heerden, J.; Grabow, W. O. K.; Ehlers, M. M. Prevalence of Vaccine-Derived Polioviruses in Sewage and River Water in South Africa. *Water Res.* **2005**, *39 (14)*, 3309–3319, https://doi.org/10.1016/j.watres.2005.05.027.
