# Supplementary figures and images for: Sampling Schemes in Poliovirus Wastewater Surveillance Studies from European Countries and Their Comparison to Other Studies: A Literature Review
Source: Microorganisms. 2026 Apr 11;14(4):861. doi: 10.3390/microorganisms14040861 (PMC13119512; doi:10.3390/microorganisms14040861)

**Figure S1:** A flow chart of the selection of studies for the literature review.

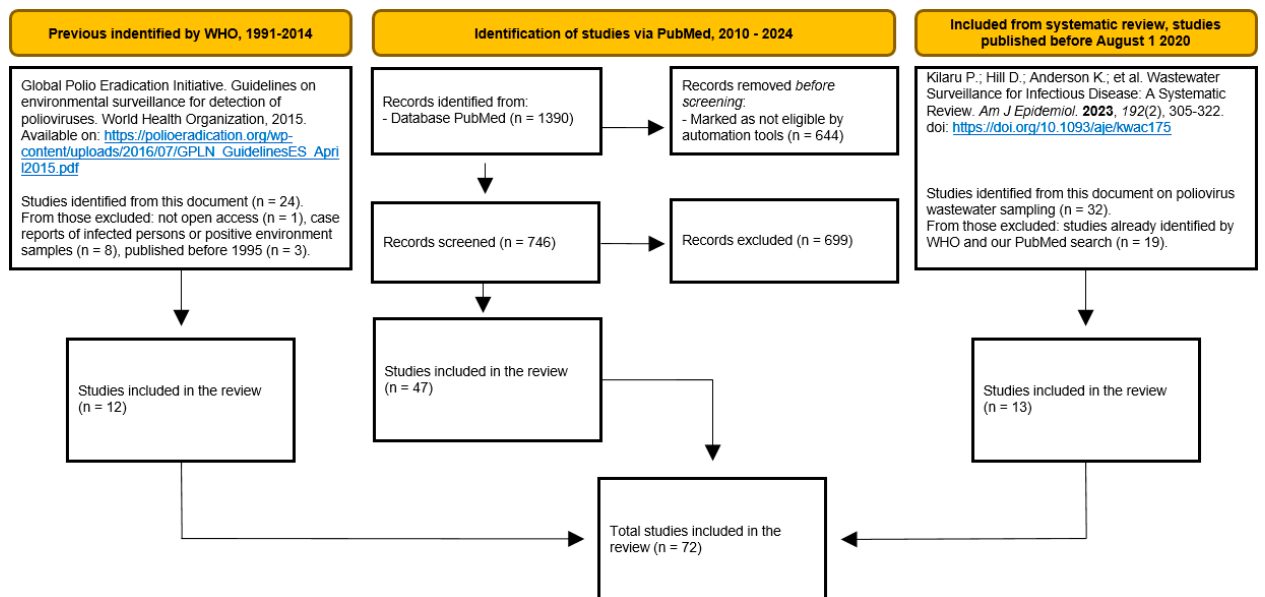

Supplement: Supplementary file 1 [file microorganisms-14-00861-s001.zip › Supplement Figure S1.pdf]
